# Male ornaments predict contact and parasite transmission between pairs of guppies, *Poecilia reticulata*

**DOI:** 10.1098/rspb.2026.1906

**Published:** 2026-11-11

**Authors:** Faith Rovenolt, Rachael D. Kramp, Vineet Nayak, Carolyn D. Tett, Catherine Wynne, Devin J. Henry, Lindsay Colgan, David Clark, Emmalina Calcaterra, Wouter van der Bijl, Jason C. Walsman, Jessica F. Stephenson

**Affiliations:** ^1^ Department of Biological Sciences, University of Pittsburgh, Pittsburgh, PA 15260, USA; ^2^ Department of Biological Sciences, University of Denver, Denver, CO 80208, USA; ^3^ Department of Zoology and Biodiversity Research Centre, The University of British Columbia, Vancouver, V6T 1Z4, Canada; ^4^ Division of Natural and Applied Sciences, Duke Kunshan University, Kunshan, 215300, People's Republic of China; ^5^ Earth Research Institute, University of California Santa Barbara, Santa Barbara, CA 93106, USA; ^6^ Department of Zoology, Stockholm University, Stockholm 106 91, Sweden

**Keywords:** parasite-mediated sexual selection, host behaviour, parasite transmission, sexually selected traits, *Gyrodactylus turnbulli*, *Poecilia reticulata*

## Abstract

Males with higher quality ornaments are often more parasite resistant and should therefore present less transmission risk to conspecifics, but this prediction has never been directly tested. Transmission risk is a product of both behaviour, dictating contact between hosts, and parasite transmissibility, dictating which contacts result in transmission. We tested how male ornament quality shapes both, using pairs of Trinidadian guppies (*Poecilia reticulata*) in which one was infected with a directly transmitted ectoparasite (*Gyrodactylus turnbulli*). Uninfected male ‘recipients’ with larger orange ornaments both initiated fewer contacts with, and received fewer contacts from, infected ‘donors’ of either sex. Male donors with larger orange ornaments and few parasites initiated the most contact, and those with large ornaments and many parasites the least. Orange ornaments also structured transmission: heavily infected donors with larger ornaments transmitted the most parasites. The sexes did not differ in their response to male ornaments, but did differ in how they transmitted parasites. Thus, more ornamented males present a considerable transmission risk, counter to prevailing hypotheses, and perhaps driving their avoidance by both sexes. Our results suggest sexually selected ornaments influence host contact behaviour and parasite transmission in complex, interacting ways with implications for sexual selection and disease dynamics.

## 1. Introduction

Mate choice has profound implications for animal evolutionary ecology [1], yet how it may be affected by parasites remains poorly understood [2]. Parasite-mediated sexual selection hypotheses propose that choosers (hereafter, females) prefer potential mates with higher quality ornaments (hereafter, males) because ornamentation advertises resistance [3]. Resistance is the ability of a host to limit the number of parasites it carries, ‘parasite load’ [4], and males with higher quality ornaments are consequently predicted to have the fewest parasites by the majority of classic hypotheses (reviewed in [5]). Preferring resistant males might benefit females either indirectly, by gaining resistance genes for offspring [6], or directly, by avoiding mating with infected conspecifics and therefore avoiding becoming infected themselves [7]. Overall, higher quality ornaments (e.g. bigger bowers, redder combs, longer tail feathers) do predict lower parasite loads, but only when parasites are transmissible during mating [5]. While this result suggests that females may reduce their probability of infection by mating with highly ornamented males, whether such mates actually transmit fewer parasites remains untested.

In general, it is unclear how sexual contact may affect the spread of parasites that can use but do not require sex to transmit [8,9]. Transmission risk for directly transmitted parasites is determined by both host behaviour—contacts with other conspecifics—and the infectiousness of the host during contact—the number of parasites transmitted [10]: ornament quality probably predicts both components. All else being equal, hosts with more contact with conspecifics are more likely to become infected or, if already infected, transmit parasites to other individuals. A male's ornament quality affects how he interacts with conspecifics [11–14]: males with higher quality ornaments probably receive more contacts from females because they are more preferred [15], potentially increasing their infection risk from females [16]. However, if females lose their preference for high-quality ornaments when infected [17,18], male ornament quality may not reliably predict infection risk from females. Further, it is unclear in most systems how females rank infection and ornament quality—uninfected females could still prefer males with higher quality ornaments, even if they are infected, because of the indirect benefits of resistance genes. Thus, how male ornament quality predicts contact between males and females in the presence of infection remains unclear.

The number of parasites transmitted during contact between hosts is controlled by many host and parasite factors, but may correlate with ornament quality. For the host, these factors include the infected host's stage of infection [19], and the number of parasites on the transmitting host [20,21]. The number of parasites that transmit is often assumed to increase with the number of parasites on the infected host, but this relationship may be nonlinear [20,21]. Parasite transmission may be density-dependent—the more parasites on the infected host, the more they compete for resources and the more likely they may be to transmit [22]. If parasite dispersal away from an infected host is density-dependent, then—as is commonly assumed and observed [23–25]—the most infected hosts would transmit more parasites and resistant hosts would transmit fewer. Under such parasite density-dependent transmission, males with higher quality ornaments may pose a low risk of transmission. However, parasite transmission may also be condition-dependent—parasites may transmit away from unfavourable host conditions in search of a more hospitable host [21,26] suggesting males with higher quality ornaments instead pose a high risk of transmission. Parasites may also be less likely to establish infections on resistant males [20], so ornaments may additionally predict the probability of parasite infection establishment on previously uninfected hosts.

Here, we tested how male ornament quality and resistance—through their impacts on contact behaviour and infectiousness—might predict the role males play in epidemic dynamics. We simultaneously measured ornamentation, parasite load, behaviour and direct parasite transmission in pairs of Trinidadian guppies (*Poecilia reticulata*) in which one was infected with *Gyrodactylus turnbulli*, a directly transmitted ectoparasite. Guppies are small, freshwater fish native to the Caribbean and northern South America with males characterized by coloured skin patches (hereafter, ornaments). These ornaments can be a wide range of colours including black, orange and iridescent. Females generally prefer more conspicuous males, with a preference for orange being the most consistent across populations [27]. In at least one population, greater orange area predicts greater resistance to *G. turnbulli*, as measured by males having fewer cumulative parasites over the course of an infection [28]. Females from this same population prefer males with larger areas of orange [29]. However, females from at least one other native population lose their preference for males with larger areas of orange when they are infected [18]. Guppies avoid infected conspecifics in the later stages of infection when transmission risk is highest [19]. Additionally, at least among female guppies, more resistant hosts transmit more parasites [21].

In this system, therefore, we expect to see a complex relationship between male ornamentation, contacts and parasites transmitted. We allowed pairs of guppies to freely interact, recording the number and directionality of contacts as well as the number of parasites that transmitted from infected ‘donor’ to uninfected ‘recipient’. Our results provide rare insight into the complex interplay of host behaviour and parasite transmission, especially in the context of sexually selected ornamentation.

## 2. Material and methods

### (a) Fish rearing

We used laboratory-bred descendants of wild guppies collected in 2012 from the Caura River in Trinidad (Universal Transverse Mercator (UTM): 20 P 6 79 527.7 m E, 11 80 376.4 m N based on WGS84 Datum; elevation 112 m). Females from this population prefer males with larger orange ornaments [29,30]. All fish were kept on a 12 : 12 light–dark cycle. Stock populations were fed daily with Tetramin tropical flake and *Artemia* sp. nauplii hatched from decapsulated eggs. Fish were kept on a recirculating system (Aquaneering) with approximately 20% daily water changes (reverse osmosis water, reconstituted with Instant Ocean, magnesium and calcium) and an average water temperature of 25°C ± 1°C.

Fish that entered the behaviour and transmission trials as donors were collected as juveniles from breeding tanks as soon as they were detected and kept in individual 1.5 l tanks. Fish were fed autoclaved pellets (following the recipe in [31]), supplemented with Viatchem (Boyd Enterprises) and Tetramin flake daily. Water was manually exchanged three times a week from the same system as stock populations.

Owing to the design of an experiment running concurrently with this one, we used recipients raised under two different conditions. Approximately half of the recipients were reared the same as the donor fish (isolation-reared; *n* = 30). The other half (stock-reared; *n* = 29) were haphazardly pulled from stock populations 2 weeks before behaviour trials and housed in 1.5 l tanks under the same conditions as the other recipients and donors until the trials began. This difference in rearing meant that the donors and isolation-reared recipients were virgins entering the behaviour trials, while stock-reared recipients were probably not. Isolation-reared male recipients also tended to have smaller areas of relative orange. We included whether or not the recipient was isolation-reared in our analyses, but it did not explain any variation in either contact behaviour or parasite transmission (see supplementary material).

### (b) Parasite husbandry

The isogenic line of the ectoparasite *G. turnbulli* (species identity confirmed with molecular methods described in [32]) used in this experiment originated from a single parasite isolated from a commercially obtained guppy in 2019. We maintained the line on groups of parasite-naive stock fish under standard conditions (hereafter, culture fish), counting the number of parasites on each culture fish biweekly (*G. turnbulli* infects guppy skin) and adding more naive fish as necessary.

### (c) Quantification of male relative orange area

Male donors were photographed the day before manual infection and recipients were photographed 1–2 days before their trial. Orange ornaments were quantified following the protocols in Morris *et al.* [33] and van der Bijl *et al.* [34]. Males were placed in an approximately 8 × 2 × 10 cm tall clear glass tank that minimized their ability to turn and placed in a 30 × 60 × 30 cm box made of white opaque plastic. The small glass tank was oriented in the box such that the 8 cm side of the tank was perpendicular to the camera. Images were captured with a Nikon D7000 camera fitted with an AF-S DX Micro-Nikkor 85 mm f/3.5G lens positioned approximately 30 cm from the tank (shutter speed: 1/640 s, aperture: f/8, International Organization for Standardization (ISO): 100). The tank was lit from above with two NEEWER NL660 Bi-Color LED Panel Lights at full white lighting. Males were photographed until at least two ‘good’ photos were taken of both his sides—‘good’ photos showed the male completely perpendicular to the camera, not turned or twisted either side-to-side or rotationally. Photos were then colour corrected using a X-Rite ColorChecker Passport Photo 2 taken under the same lighting and camera conditions. Photos were processed with a semi-automated pipeline that masked the fish body (excluding gonopodium and dorsal fin), and extracted the percent of body area covered with orange ornaments from each image [34]. Orange ornament area was therefore the percent body area covered by orange ornaments, first averaged across the two images of a male's side, and then across both sides. Ornament quantification was performed by an observer unaware of the treatment, and in random order.

### (d) Manual infection of donors

We recorded the body length of donors the day they were manually infected. Donor fish were manually infected by anaesthetizing them with tricaine methanesulfonate (MS222; 4 g l^−1^) and placing them under a dissecting scope next to a heavily infected euthanized culture fish until approximately two parasites transmitted (mean ± s.e.m. = 2.33 ± 0.108). Donor fish parasite loads were counted under dissecting scope the day before their trial to determine the number of parasites on the donor that could potentially transmit to the recipient.

### (e) Recipients and pairing

We recorded the body length of recipients the day before their trial. Donors and recipients were haphazardly paired to create three treatment groups based on donor and recipient sex: female donor and male recipient (F > M pairs; *n* = 14), male donor and female recipients (M > F pairs; *n* = 14), and male donor and recipients (M > M pairs; *n* = 14). We controlled for the size of paired fish in our analyses.

### (f) Behaviour and transmission trials

Trials were conducted in five batches between 13 November 2021 and 11 December 2021, between 10.00 and 17.00 on trial days. Donors were on their 10th day of infection going into the trials, which represents sufficient time for infections to develop and vary between fish [21], and limits parasite-induced mortality [35]. All fish were at least 90 days old before entering the behaviour trials, and thus sexually mature [36]. To conduct the trials, 6 l tanks were filled with approximately 1.5 l system water—a depth of approximately 3 cm—to minimize fish overlapping in the recording. Tanks were filmed at 10 frames per second from above using a FLIR Blackfly USB3 camera, and lit with diffuse LED lights. This set-up was in a room separate from fish husbandry facilities, and the tank was visually isolated using white boards. At the beginning of each trial, the recipient was isolated in a transparent acetate tube inside the tank, while the donor swam freely throughout the tank. Donor and recipient could see each other. After a 20 min acclimation period, the acetate tube was removed via a pulley system to allow the donor and recipient to interact freely. Their behaviour was recorded for 10 min. At the end of the recording, the donor and recipient were immediately decanted from their tank and checked for transmission. Tanks were sprayed with 70% ethanol, rinsed thoroughly and refilled between trials.

### (g) Quantifying transmission

Transmission was verified by discovering at least one parasite on the recipient under a dissecting scope. If transmission occurred, donor and recipient were returned to their separate individual tanks. If transmission did not occur post-trial, donors and recipients were co-housed and re-checked for transmission daily until either transmission occurred or there were no parasites left on the donor. Three pairs did not transmit any worms during the behavioural trial. As this is insufficient variation to analyse directly, we scored these pairs as having transmitted 0 worms on day 1 of transmission, and focus our analysis on the number of worms transmitting. Fish were used once and then euthanized.

### (h) Video processing and contact initiation

All videos were processed with TRex [37], a tracking software that assigns unique IDs to individuals. We manually linked TRex-generated IDs to donor and recipient identity by tracking the location of recipients from their release from the acetate tubes to the beginning of ID assignment by TRex manually. We defined a contact as any frame in which the TRex-identified centroids of the donor and recipient came within 3.5 cm of each other.

Contacts were further refined by evaluating which fish was initiating contact. Once the centroids of the fish were within 3.5 cm (approximately 2 body lengths), contact initiation was attributed to a fish if, in the next frame, its centroid moved closer to the other fish's centroid into a 90° window centred on the other fish's centroid. The number of frames of initiated contacts was then divided by the total number of frames in the video to generate the proportion of time initiating contact for each fish. We used frames rather than seconds because of slight differences in duration between videos. This measurement is unitless, ranges from 0 to 1 (though in practice is much less than 1), and is calculated per pair of fish.

Thus, donor initiation is the number of frames in which the donor moved towards the recipient, while both were within 3.5 cm, divided by the total number of frames of the video. Similarly, recipient initiation is the number of frames the recipient moved towards the donor divided by the total number of frames. It is important to note that donor and recipient initiation are not mutually exclusive—both fish could initiate contact by both moving towards each other. Additionally, not every contact was attributed to either donor or recipient—the fish may have been within 3.5 cm of each other for a frame but moving away from each other or obliquely passing each other.

While contact initiation begins to unravel the directionality of contacts, it does not fully distinguish or identify the exact nature of the interactions between two hosts. It does not, for example, identify when a male is courting a female versus attempting a sneak copulation with her [27]. Additionally, it does not tell us the duration of interactions—several short bouts of interaction could result in the same amount of contact initiation as one long interactive period. However, for pairs with males and females, this does approximate a no-choice preference test (as opposed to a dichotomous test) and thus we use a female's initiation of contact with a male as a proxy for preference [27].

### (i) Statistical analyses

All analyses were performed with R version 4.4.2 [38], and all data and code used are presented in the supplementary material. We used linear models (LMs) in base R, negative binomial generalized linear models using *MASS* [39], visualized model fit and performance with the *DHARMa* package [40], and plotted figures with *visreg* [41] and *ggplot2* [42].

Our analyses address three questions: (i) Do recipient ornaments predict contact? (ii) Do donor ornaments predict contact? (iii) Do ornaments predict transmission? We split the data into subsets to understand how recipient ornaments structure contact and transmission (using only pairs in which the recipient was male: F > M and M > M), and how donor ornaments structure contact and transmission (using only pairs in which the donor was male: M > F and M > M). We therefore use six separate models to address our three questions using two data subsets.

Our overall approach and some details are the same across all six models. We first visually inspected the distributions and correlations between our variables in each subset (see supplementary material). We included as explanatory variables in all models the number of parasites on the donor, the standard length of the donor, the standard length of the recipient, whether the recipient was isolation-reared, and the Julian date of the behaviour trial. We describe below further model-specific explanatory variables. For each model, we initially checked the variance inflation factors (VIFs) of the main effects model (excluding interactions) using *car* [43]: no VIF was over 2*.* We then included two-way interactions about which we had *a priori* hypotheses (described below). Given our modest sample size, we prioritized parsimony to avoid overfitting and performed stepwise model simplification using the *drop1* function, retaining any variables that improved model fit, regardless of their significance. We provide all code and plots of model fits in the supplementary material.

#### (i) Do recipient ornaments predict contact?

In models 1 and 2, we tested how recipient ornaments and other variables explained variation in recipient contact initiation rate, and donor contact initiation rate, respectively, using linear models. As well as those listed above, we included as explanatory variables the recipient's relative orange area (‘recipient orange’), the sex of the donor, and all two-way interactions between recipient orange, sex of the donor and the number of parasites on the donor.

#### (ii) Do donor ornaments predict contact?

In models 3 and 4, we tested how donor ornaments and our other variables explained variation in recipient contact initiation rate, and donor contact initiation rate, respectively, again using linear models. We included the donor's relative orange area (‘donor orange’), the sex of the recipient, and all two-way interactions between donor orange, the sex of the recipient and the number of parasites on the donor.

#### (iii) Do ornaments predict transmission?

In models 5 and 6, we tested how the number of parasites transmitting could be predicted using recipient and donor ornaments, respectively, using negative binomial generalized linear models. During preliminary analysis, we found that donor and recipient contact initiation rates were moderately correlated (*r* = 0.42–0.5), and models containing both variables simultaneously provided little improvement in fit over models containing recipient initiation rate alone. We therefore included recipient, but not donor, contact initiation in models 5 and 6.

We included the same explanatory variables as in the contact initiation analyses because, independent of how they affect contact initiation, they could impact the number of parasites transmitted. For example, the length of the fish could impact the number of parasites transmitted outside of their impact on contact initiation as larger fish have a greater surface area from and to which parasites might transmit. Additionally, since contact initiation is defined at a set distance between centroids, the lengths of the fish could impact how close a pair actually come within that between-centroid distance. We also included relative orange area (of the recipient in model 5 and donor in model 6) as, if ornaments signal resistance, they may affect both contact behaviour and the transmission of parasites between fish during contact (i.e. ornament quality might predict both contact and parasite transmission given contact). We included fish sex (of the donor in model 5, and recipient in model 6) as sex may affect how the fish interact and thus transmission. We included all two-way interactions between ornament, sex, number of parasites on the donor and recipient contact initiation rate.

## 3. Results

### (a) Do recipient ornaments predict contact?

Recipients with larger relative areas of orange both initiated fewer contacts with donors (figure 1A, model 1: F = 5.2; *p* = 0.033), and received fewer contacts from donors (figure 1B, model 2: F = 14.4, *p* < 0.001). We also found that recipient contact initiation depended on the sex of the donor and the donor's parasite load (figure 2A, model 1: F = 4.7, *p* = 0.04). *Post hoc* tests show that neither of these slopes differ from 0, however. The length of the donor remained in the final model 1, but did not significantly predict recipient contact initiation. No other terms remained in the final model 2.

**Figure 1.**
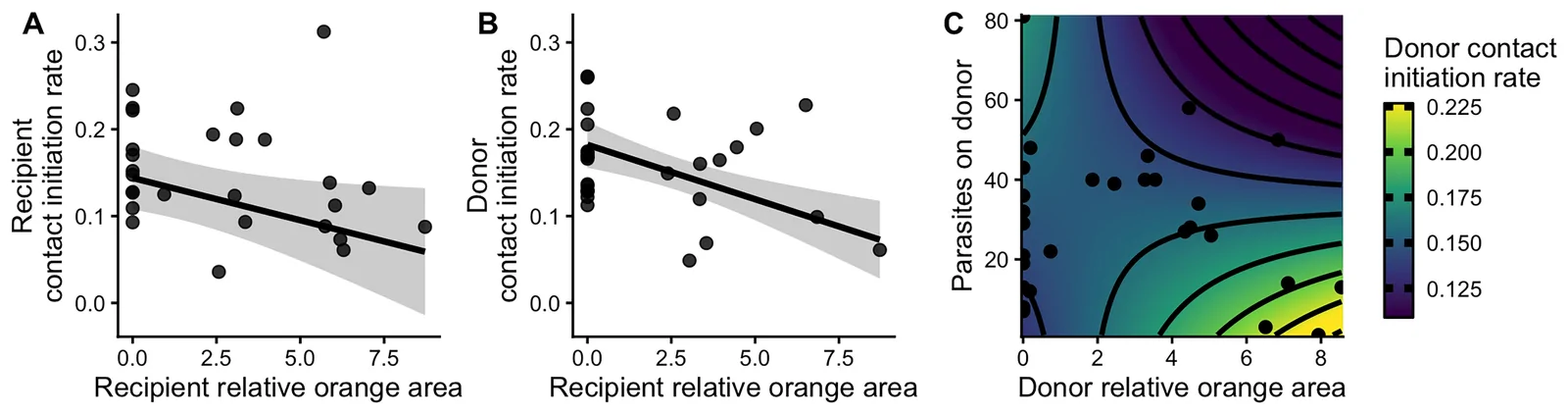
Ornaments predict contact. Uninfected ‘recipients’ with larger relative areas of orange both initiated less contact with infected ‘donors’ (A), and received less contact from them (B). Lines give the model fits, and shading the 95% confidence interval (A: model 1; B: model 2), and points give the raw data. (C) Filled contour plot, with colour indicating model-predicted donor contact initiation rate (model 4; light, more yellow = greater contact initiation; darker, more purple = lower contact initiation). Thick lines represent levels of contact initiation as indicated on the colour bar, and points represent raw data.

**Figure 2.**
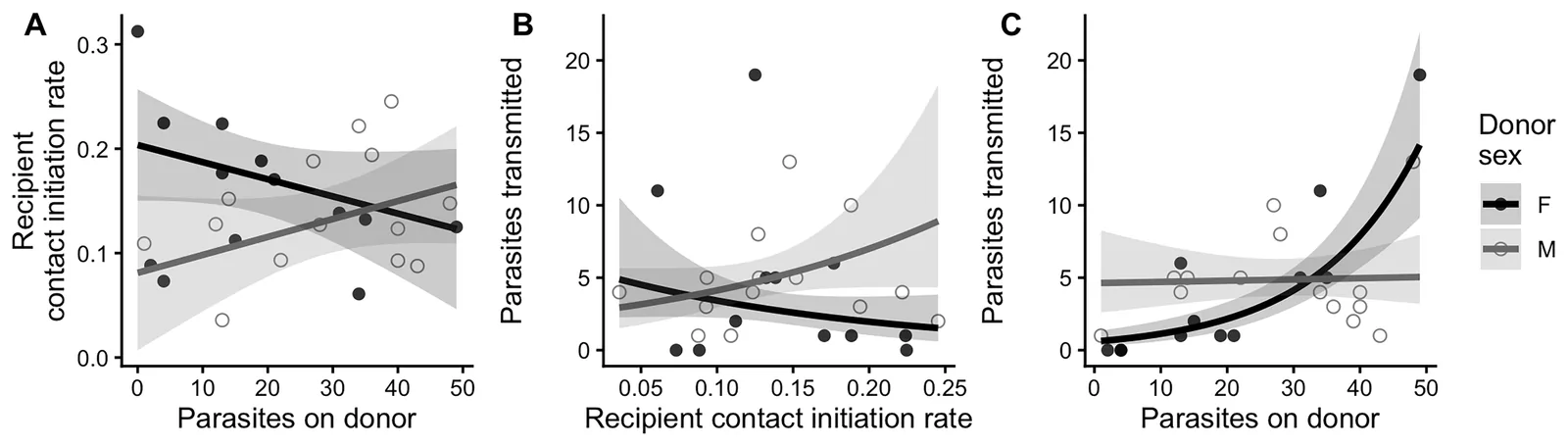
Donor sex affects contact patterns and transmission. (A) Recipient contact initiation rate depended on the number of parasites on the donor, and the sex of that donor (neither slope differs significantly from 0; model 1). (B) Recipient contact initiation rate significantly predicted the number of parasites it received, again depending on donor sex (again, neither slope differs significantly from 0; model 5). (C) The more parasites a female donor had, the more she transmitted, but parasite load did not predict the number of parasites transmitting from male donors (female donor, but not male donor, slope differs significantly from 0; model 5). On all panels, lines give the model fits, shading the 95% confidence intervals and points the raw data.

### (b) Do donor ornaments predict contact?

Donor ornaments did not explain significant variation in recipient contact initiation rate, nor did any other variable in model 3: the final model included none of our predictors. However, donor relative orange area interacted with donor parasite load to predict the donor's own contact initiation rate (figure 1C, model 4: F = 4.46, *p* = 0.045): donors initiated contact with recipients at the highest rate when they had low parasite loads and large orange ornaments. No other terms remained in the final model 4.

### (c) Do ornaments predict transmission?

The recipient's relative orange area did not predict transmission directly, but may have done so indirectly: recipient orange predicts recipient contact initiation rate, which in turn predicted the number of parasites received. Recipients who initiated more contact with male donors received more parasites, whereas those who initiated more contact with female donors received fewer (figure 2B, model 5: $\chi^{2}$ = 4.7, *p* = 0.03; *post hoc* tests show neither slope differs significantly from 0). Model 5 also indicated that once infected, the sexes pose quite different transmission risk to conspecifics: female donors transmitted more worms as their own parasite loads increased, whereas male donor parasite load did not predict the number of worms he transmitted (figure 2C, model 5: $\chi^{2}$ = 20.12, *p* < 0.0001; *post hoc* tests show that the female donor slope, but not the male, differs significantly from 0). Larger donors also transmitted more parasites (model 5: $\chi^{2}$ = 12.72, *p* < 0.001). The two-way interaction between recipient contact initiation rate and the donor's parasite load remained in the final model 5, but did not explain significant variation in number of worms transmitted.

Donor relative orange area, however, did predict transmission, depending on his parasite load (figure 3, model 6: $\chi^{2}$ = 7.74, *p* = 0.005): donors with larger orange ornaments posed a higher risk of transmission than less ornamented males, unless they were carrying very low parasite loads. Model 6 also found that larger donors transmitted more parasites ($\chi^{2}$ = 7.23, *p* = 0.007). No other terms remained in the final model 6.

**Figure 3.**
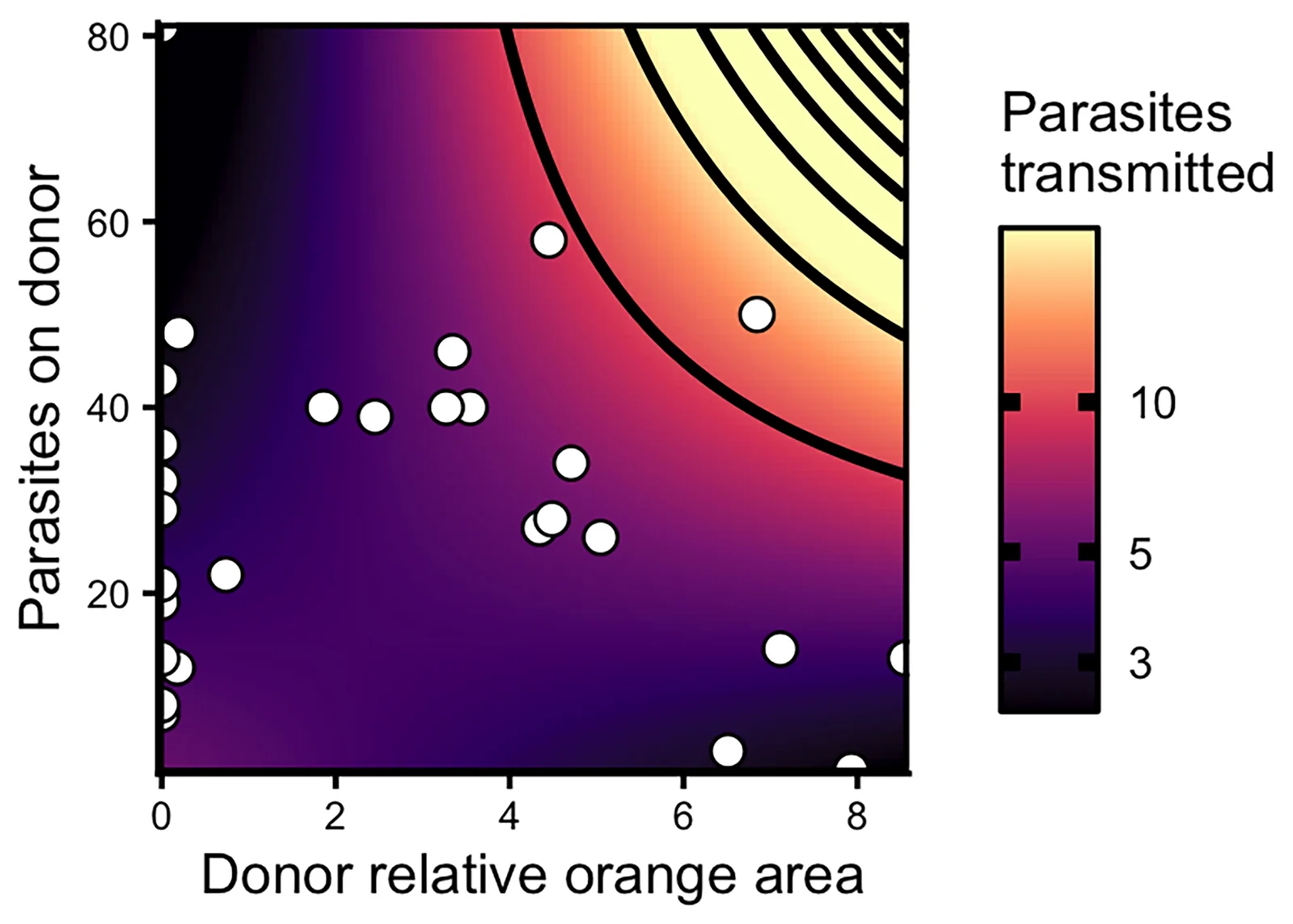
Ornaments predict transmission. Filled contour plot, with colour indicating model-predicted number of parasites transmitted (model 6; light, more yellow = more parasites transmitted; darker, more purple = fewer parasites transmitted). Thick lines represent levels of parasites transmitted as indicated on the colour bar, and points represent raw data.

## 4. Discussion

We found that ornament quality predicted both contact and the transmission of parasites between hosts. First, uninfected recipient males with larger orange ornaments both initiated and received fewer contacts from infected donors of either sex. Infected donors with larger orange ornaments also initiated fewer contacts with uninfected recipients of either sex, unless they had very low parasite loads, in which case they initiated more contact than any other donors. The pattern of donor contact initiation matches almost perfectly the number of parasites they transmitted (compare figure 1C to figure 3): donors with the largest orange ornaments and heaviest parasite loads transmitted the most parasites, despite their low contact initiation rates. Recipient contact initiation may help explain this counterintuitive pattern: recipients initiated more contact with heavily infected male donors, and the more contact recipients initiated with male donors the more parasites they received. While initiating more contact with more heavily infected male donors seems like risky behaviour, our results also suggest that transmission from males is not predicted by parasite load. Transmission from males may instead be best predicted by his ornament quality, potentially explaining the avoidance of even uninfected males with a larger relative orange area in the presence of parasites. Our results therefore indicate that males with high-quality ornaments represent a high risk of transmission, counter to predictions, with implications for our understanding of how parasites may mediate sexual selection. We discuss how our results relate to previous work in this system and the implications for the field more broadly.

Uninfected males with larger orange ornaments in general experienced lower contact with infected fish than did those with smaller ornaments, mediated by the behaviour of both the infected and uninfected fish in a pair. Uninfected males with larger orange ornaments initiated fewer contacts with infected fish of either sex than did those with smaller orange ornaments. That is, these males invested in behavioural resistance, despite the fact that larger orange ornaments are associated with greater physiological resistance in this population [28]. This contrasts with the idea that animals should trade-off these resistance strategies, which has gained some empirical support from this [44] and other systems [45,46]. Resistance does carry costs for males in this system [47], so short-term avoidance may well be cheaper even for these males. Additionally, males with larger orange ornaments—and thus better prospects of future reproduction [29,30]—may have more to lose than those with smaller ornaments in terms of future reproductive value, so their stronger infection avoidance behaviour is in line with the asset protection principle [48].

Low contact with and by males with more orange may be driven by the correlation between donor and recipient contact initiation rates. While guppies avoid cues of infection [19,49], it is unclear to what extent they can detect infection and assign it to individual conspecifics. Donors had more information that infection was present, and indeed recipient relative orange area appears to affect contact initiation rate more strongly among donors than recipients. It is possible that in the presence of infection cues—even those emitted by themselves—guppies avoid males with larger orange ornaments as a risk-sensitive infection-avoidance strategy.

Our clear evidence of avoidance of uninfected males with larger orange ornaments also represents an infection-induced reversal of typical female preferences. In general, while parasite-mediated sexual selection hypotheses usually assume the presence of parasites in the system, they do not consider how choosers themselves becoming infected may affect their preferences, or the choices they make [50,51]. While we did not explicitly test female preference in the absence of infection, we used fish descended from a wild population in which females prefer males with larger orange ornaments [29,30], and female guppy preference evolves slowly and appears stable through time [51]. Our result is also consistent with those of López [18], who showed that *Gyrodactylus* spp.-infected females from the Paria performed fewer receptive ‘glides’ for attractive males (i.e. those with more intense orange and higher display rate) than uninfected females. Across systems, parasitized receivers in a mate choice context may become less choosy [17,52–54], choosier [55,56] or show no difference [57–60]: the drivers and implications of this variation in effects of infection on chooser behaviour for sexual selection and disease dynamics warrant further investigation.

In general, though there is substantial evidence that parasites affect animal cognition [61], how infection affects the receivers of communication is chronically understudied [50] and has substantial implications for how parasites may mediate sexual selection. In our system, avoidance of males with larger areas of orange, either by infected females or females around infected males, could reduce sexual selection for males with larger areas of orange; this in turn could increase parasite prevalence by decreasing the proportion of orange, resistant males in the population [51]. However, female preference could also reinforce honesty in sexual signalling if males with larger ornaments only mate when they are uninfected [62]. Importantly, we found no difference between male and female donors in their avoidance of male recipients with larger orange ornaments: how ornaments mediate intrasexual contact and parasite transmission is an important and currently largely overlooked aspect of parasite-mediated sexual selection.

As well as recipient ornament quality predicting contact behaviour, we found that donors with larger orange ornaments initiated fewer contacts as their parasite loads increased. This pattern may be indicative of somatic maintenance, as higher quality males allocated energy away from social or mating behaviours and towards dealing with infection as an investment in future mating opportunities [63]. Male guppies invest more in mating behaviours when paired with a receptive female [64], and thus a lack of initiation from females combined with the costs of infection [65] may have driven the decrease in contact initiation by males with larger orange ornaments and higher parasite loads. Further, resistant males with larger orange ornaments and low parasite loads may have been close to clearing their infections, so this result may be analogous to that of López [66] in which males that had acquired resistance and recovered from infection with *Gyrodactylus* spp. had higher display rates.

Notable in our results on how ornament quality mediates contact behaviour is the lack of sex difference. How guppies interact often depends on their sex and male ornament quality. Males may court females or engage in sneak mating [27], and males with larger orange ornaments are often more likely to court [11,12]. Additionally, female responses to male displays—ranging from glide responses to aggression—often depend on his ornament quality [27]. Male–male interactions also depend on ornamentation, including relative orange area [14]. However, in our short trials, it is possible that infection avoidance—rather than mating—was the most salient pressure, and both sexes appear to respond in the same way to cues of infection [19].

As well as ornament quality affecting contact between fish, we found evidence that it predicts the number of parasites transmitting between them. Again regardless of the sex of the recipient, more parasites on and greater orange area of the male donor were associated with more parasites transmitted. This result is consistent with parasites experiencing males with larger areas of orange as more hostile hosts, inciting parasites to transmit when competing with other parasites, supporting both parasite density- and host condition-dependent transmission [22,26]. Indeed, parasite behaviour may partially explain how these males with relatively large orange ornaments and relatively high parasite loads initiated the fewest contacts but still transmitted the most parasites [67].

We found that, as previously reported, more heavily infected female donors transmit more parasites [21], but the same is not true of male donors: parasite load is a poor predictor of the transmission risk they pose. Uninfected male recipients initiated fewer contacts with heavily infected female donors, and more contact with heavily infected male donors. Thus, recipients in mixed sex pairs, but not male pairs, initiated more contact with donors that transmitted fewer parasites, consistent with previous findings that guppies engage in risk-sensitive contact behaviour [19]. Lower initial parasite loads can lead to lower peak loads [22] and decreased likelihood of parasite establishment [21]; thus, perhaps males with larger orange ornaments, through their lower rate of contact initiation with highly infectious females, are less likely to become infected from between-sex interactions.

Field survey data and a theoretical model have recently demonstrated that female preference for male ornaments may importantly impact disease dynamics in this system in nature [51]. The model found that the effect of female preference on disease dynamics was mostly mediated through females producing offspring with more resistant males, increasing population-level resistance through successive generations. Contact with more resistant, more ornamented males did not result in less transmission because more contact overwhelmed the resistance of such males, and they became ‘sexy superspreaders’ [9,16,51]. Our findings complement this insight: males with larger (i.e.,higher quality) orange ornaments pose a substantial transmission risk once infected, so direct benefits alone may not be sufficient to drive sexual selection in this system.

More broadly, our work highlights that the understudied nexus of sexual selection and epidemiology has important implications for the ecology and evolution of hosts and their parasites. Understanding contact patterns between hosts is crucial to understanding infectious disease dynamics, and social network analysis has provided some insights upon which our work builds. Reproduction is an important driver of contact and, in the absence of parasites, ornament quality often predicts host social status [68,69] with individuals with higher quality ornaments often occupying key positions in social networks [70]. As indicated by our results, the presence of parasites can cause social network rewiring [71–73], but social network position does predict infection probability in many systems [74–76]. Our study integrates across this previous work to show that ornaments importantly structure both contact in the presence of parasites, and the transmission of those parasites. Additionally, we show that parasites mediate how ornaments predict contact between hosts, and potentially thus mate choice. These patterns have evolutionary implications: mate choice [1] and parasite infection [4] shape host evolution, and transmission shapes parasite evolution [8,16,20]. Further clarifying how sexual selection and epidemiology impact one another will thus likely yield far-reaching benefits.

## Supplementary Material



## Data Availability

Our data and code are in the supplementary material. Supplementary material is available online [77].
