# Supplementary material for: Male ornaments predict contact and parasite transmission between pairs of guppies, Poecilia reticulata
Source: Proc Biol Sci. 2026 Nov 11;293(2082):20261906. doi: 10.1098/rspb.2026.1906 (PMC13751120; doi:10.1098/rspb.2026.1906)
Supplement: Supplementary file 1 [file rspb.2026.1906.zip › rspb.2026.1906/rspb20261906_si_002.pdf]

Supplemental Material for *Male ornaments predict contact and parasites transmitted between host pairs*

Faith Rovenolt Rachael D. Kramp Vineet Nayak Carolyn Tett
Catherine Wynne Devin Henry Lindsay Colgan David Clark
Emmalina Calcaterra Wouter van der Bijl Jason C. Walsman
Jessica F. Stephenson

published in Proceedings of the Royal Society B, doi:10.1098/rspb.2026-1906

Contents

1 Introduction **1**
1.1 Data Subsets, Descriptions, & Visualization 2
1.1.1 All Trial Pairs 2
1.1.2 Tests of the importance of the recipient's relative orange area 5
1.1.3 Tests of the importance of the donor's relative orange area 6

2 Do male ornaments structure contact between pairs? **6**
2.1 Do recipient ornaments structure contact? 6
2.1.1 Model 1 - Does recipient orange predict contacts initiated by those recipients? 6
2.1.2 Model 2: Does recipient orange predict contact initiated by donors? 8
2.2 Do donor ornaments structure contact? 10
2.2.1 Model 3 - Does the orange on the donor structure contact initiated by the recipients? 10
2.2.2 Model 4 - Does donor orange structure contact initiated by the donors? 11

3 How does orange structure transmission? **13**
3.1 Model 5: Do recipient ornaments structure transmission? 13
3.2 Model 6: Do donor ornaments structure transmission? 17

4 R and Library Versions **20**

5 References **21**

1 Introduction

In this paper, we examine how a color ornament of male laboratory-bred guppies *Poecilia reticulata* predicts contact initiation in pairs of guppies where one is infected (hereafter the "donor" fish) and the other is not (hereafter the "recipient" fish) and then whether contact initiation and ornamentation predicts the number of parasites transmitted between the pair. Specifically, we use relative orange area, which has been previously found to predict resistance (Stephenson et al. 2020). First, we describe and visualize our data across the data subsets. Next, we present our contact initiation models followed by our parasite transmission models.

1.1 Data Subsets, Descriptions, & Visualization

1.1.1 All Trial Pairs

We use data provided in the file `Rovenolt_pairs_data.xlsx`, which contains the following variables about the pairs and fish in the pairs:

Table S1: README of the variables used in our analysis

...1	Variable Name	Variable Description
1	DONORID	The ID assigned to the donor fish in the behavior trial
2	RECIPID	The ID assigned to the recipient fish in the behavior trial
3	DONORWORMS	The number of parasites on the donor before entering the behavior trial
4	TRIALDATEJULIAN	The Julian date of when the behavior trial was conducted
5	DONINITRATE	The number of frames the donor was initiating contact divided by the total number of frames
6	RECINITRATE	The number of frames the recipient was initiating contact divided by the total number of frames
7	TRANSWORMS	The number of parasites transmitted form the donor to recipient; NA if no parasites transmitted
8	TRANSWORMS0	The number of parasites transmitted form the donor to recipient; NA if no parasites on the donor, 0 if no parasites transmitted
9	DAYTRANS	The experimental day on which transmission occurred.
10	SEXDON	The sex of the donor (M = male, F = female)
11	ORANGEAREADON	The relative orange area of the donor fish; NA if female
12	LENGTHDON	The donor's length in mm
13	ISOREAREDREC	Whether the recipient was reared in isolation (Y = yes, N = no)
14	SEXREC	The sex of the recipient (M = male, F = female)
15	ORANGEAREAREC	The relative orange area of the recipient fish; NA if female
16	ORANGER	Across all of the analyses, the orange values for M>F come from the male donors, and F>M, orange values come from the male recipients. In M>M pairs: *ORANGER:* The orange value comes from the recipient.
17	ORANGED	Across all of the analyses, the orange values for M>F come from the male donors, and F>M, orange values come from the male recipients. In M>M pairs: *ORANGED:* The orange value comes from the donor.
18	LENGTHREC	The recipient's length in mm
19	DIFFLENGTH	The difference in length between the recipient and the donor (donor - recipient).
20	PAIRTYPE	M>M is male donors and male recipients; M>F is male donors and female recipients; F>M is female donors and male recipients.

Here is a summary of the sizes of fish used in our experiments.

Table S2: Body length of male and female donors

SEXDON	N	Mean ± SD	Range
F	14	15.3 ± 0.7	14.1–16.5
M	28	15.3 ± 1.0	13.5–17.6

Table S3: Body length of male and female recipients

SEXREC	N	Mean ± SD	Range
F	14	15.8 ± 1.9	12.3–19.2
M	28	15.2 ± 1.7	11.9–19.9

Table S4: Difference in length between donors and recipients

PAIRTYPE	N	Mean ± SD	Range
F>M	14	-0.3 ± 2.0	-4.1–3.2
M>F	14	-0.3 ± 2.2	-3.2–2.9
M>M	14	0.2 ± 1.8	-3.0–4.1

We use pairwise plots to check for correlations between our variables of interest.

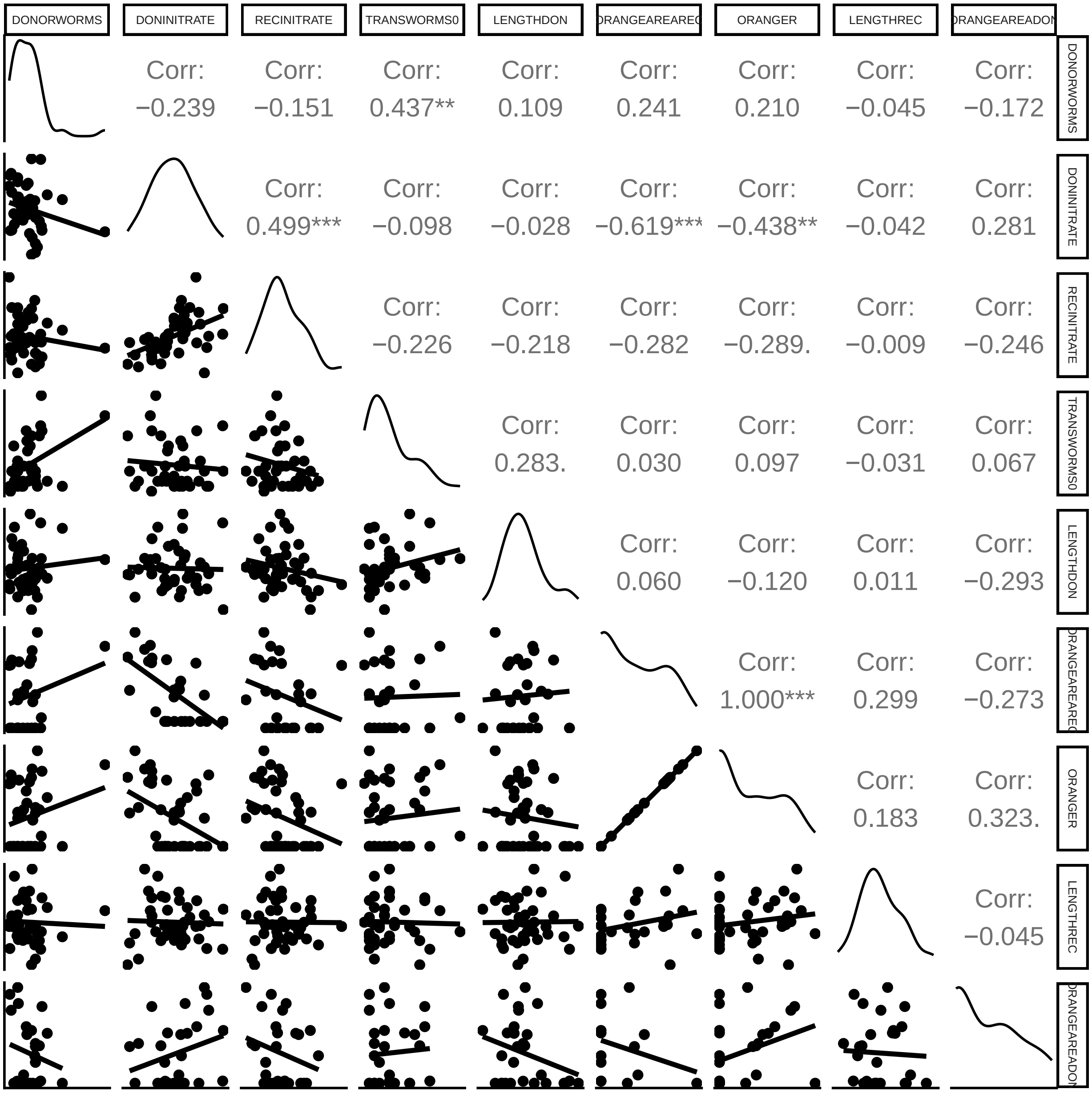

1.1.2 Tests of the importance of the recipient's relative orange area

For analysis of how a recipient's relative orange area structures contact and transmission, we subset the data to only those pairs in which the recipient was male (and thus, ornamented). We also remove one pair in which the donor's infection intensity was a significant outlier.

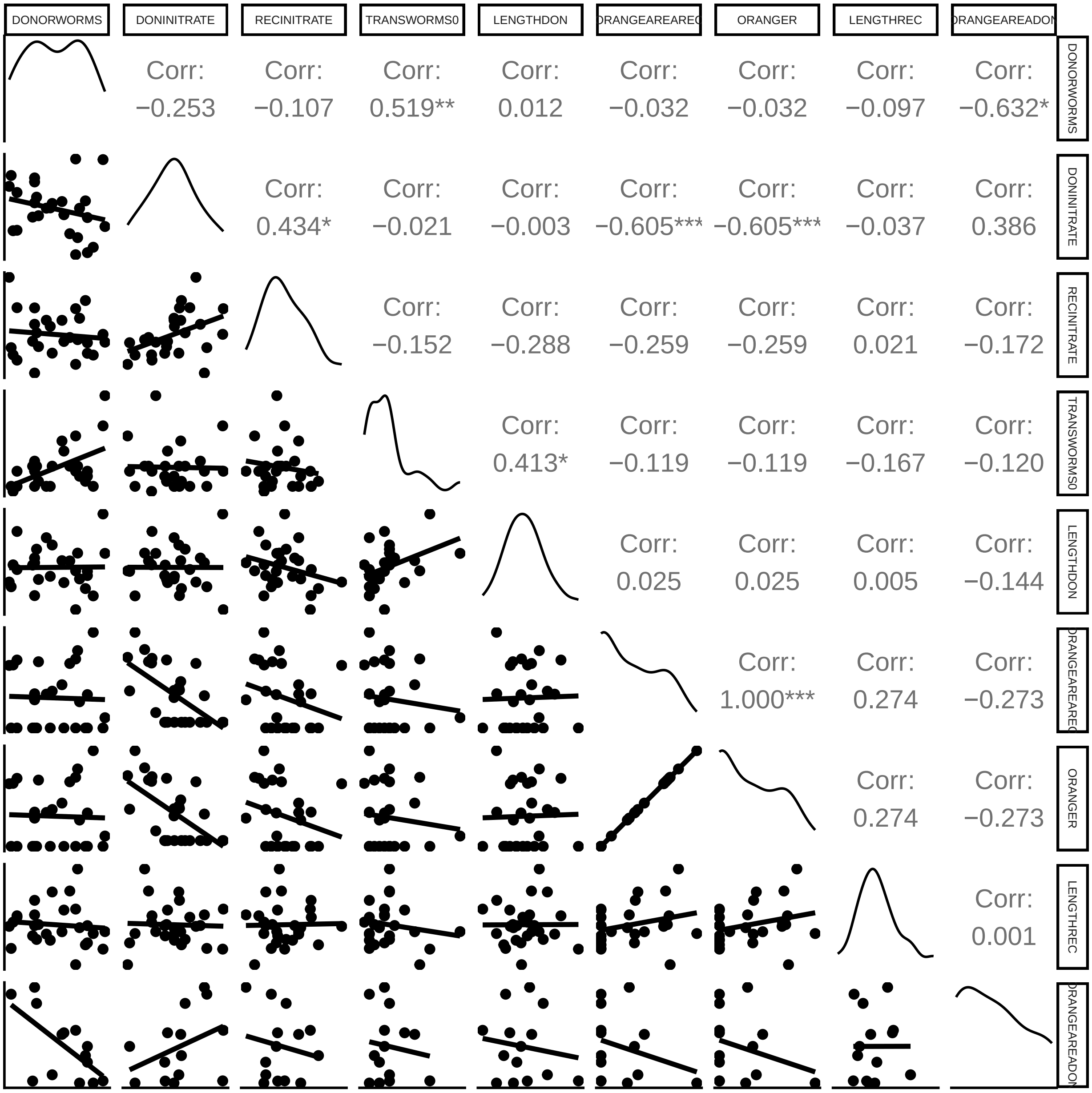

1.1.3 Tests of the importance of the donor's relative orange area

For analysis of how a donor's relative orange area structures contact and transmission, we subset the data to only those pairs in which the donor was male (and thus, ornamented).

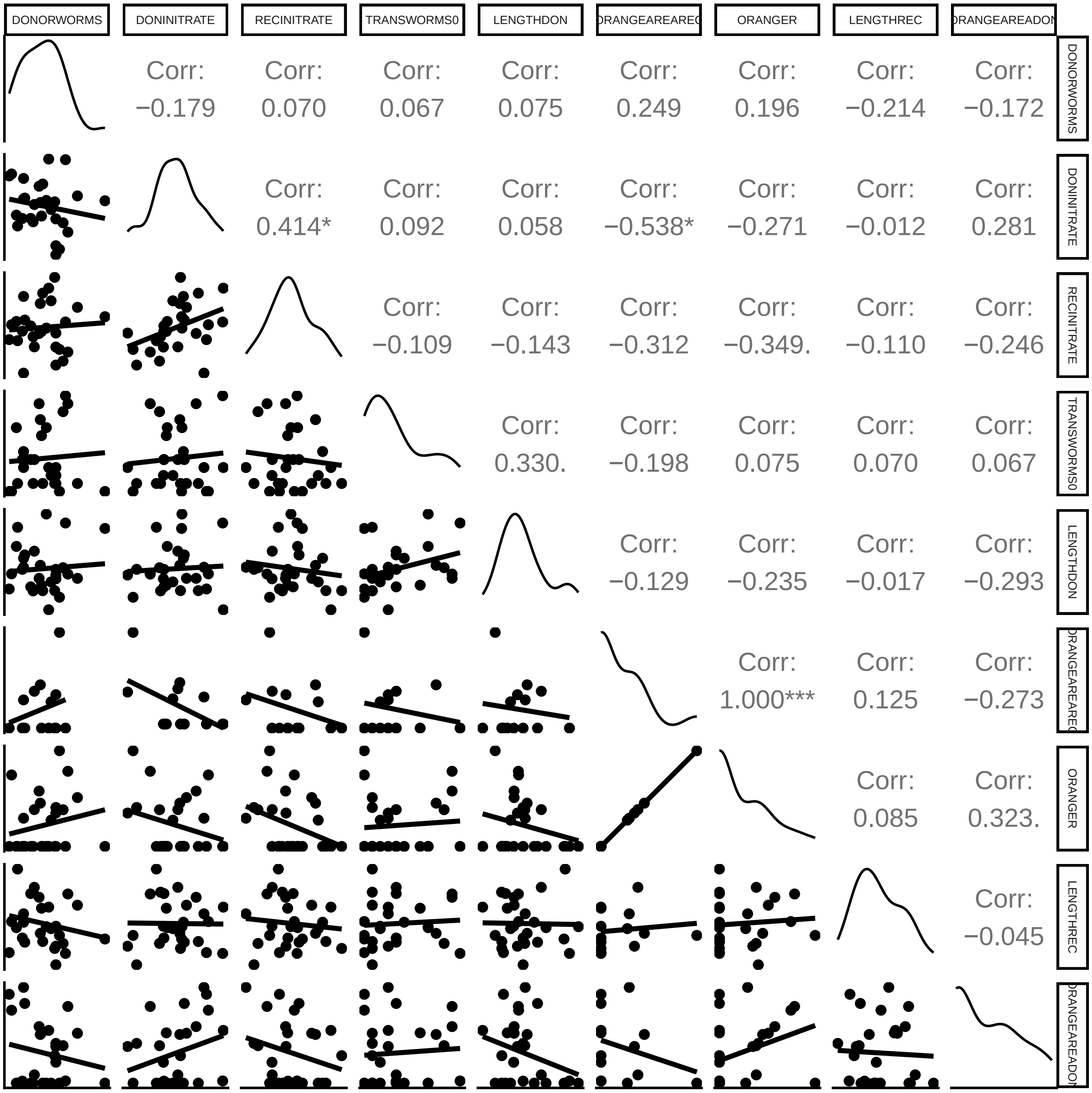

2 Do male ornaments structure contact between pairs?

2.1 Do recipient ornaments structure contact?

2.1.1 Model 1 - Does recipient orange predict contacts initiated by those recipients?

Yes, male recipients with larger orange ornaments initiate fewer contacts. Recipients also initiate more contacts with more heavily infected females, and less contacts with more heavily infected males.

```
#main effects model to check variance inflation factors.
m1m <- lm(RECINITRATE ~
```

```r
   +LENGTHDON
   +LENGTHREC
   +ISOREAREDREC
   +TRIALDATEJULIAN
    +ORANGER
   +SEXDON
   +DONORWORMS,
  data = dfr2
)
car::vif(m1m)
```

```
##       LENGTHDON       LENGTHREC    ISOREAREDREC TRIALDATEJULIAN         ORANGER
##        1.080424        1.169119        1.625011        1.164438        1.737257
##          SEXDON      DONORWORMS
##        1.457674        1.159668
```

```r
m1 <- lm(RECINITRATE ~
   +LENGTHDON
   #+LENGTHREC
    #+ISOREAREDREC
   #+TRIALDATEJULIAN
    +ORANGER
   +SEXDON
   +DONORWORMS
   #+ORANGER:SEXDON
   #+ORANGER:DONORWORMS
   +SEXDON:DONORWORMS,
  data = dfr2
)

drop1(m1)
```

```
## Single term deletions
##
## Model:
## RECINITRATE ~ +LENGTHDON + ORANGER + SEXDON + DONORWORMS + SEXDON:DONORWORMS
##                   Df Sum of Sq      RSS     AIC
## <none>                         0.064906 -150.83
## LENGTHDON          1 0.0091284 0.074035 -149.28
## ORANGER            1 0.0160744 0.080981 -146.85
## SEXDON:DONORWORMS  1 0.0145822 0.079488 -147.35
```

```r
Anova(m1)
```

```
## Anova Table (Type II tests)
##
## Response: RECINITRATE
##                     Sum Sq Df F value  Pr(>F)
## LENGTHDON         0.009128  1  2.9534 0.10040
## ORANGER           0.016074  1  5.2008 0.03312 *
## SEXDON            0.008303  1  2.6862 0.11611
## DONORWORMS        0.000002  1  0.0007 0.97895
## SEXDON:DONORWORMS 0.014582  1  4.7180 0.04145 *
## Residuals         0.064906 21
## ---
```

```
## Signif. codes:  0 '***' 0.001 '**' 0.01 '*' 0.05 '.' 0.1 ' ' 1
```

```
#visreg(m1, "DONORWORMS", by="SEXDON")
#visreg(m1, "ORANGER", scale="response", partial=T)


emtrends(m1, pairwise ~ SEXDON, var = "DONORWORMS")
```

```
## $emtrends
##  SEXDON DONORWORMS.trend      SE df  lower.CL upper.CL
##  F              -0.00164 0.00107 21 -0.003873  0.00059
##  M               0.00172 0.00111 21 -0.000593  0.00403
##
## Confidence level used: 0.95
##
## $contrasts
##  contrast estimate      SE df t.ratio p.value
##  F - M    -0.00336 0.00155 21  -2.172  0.0414
```

```
simulateResiduals(fittedModel = m1, plot = T)
```

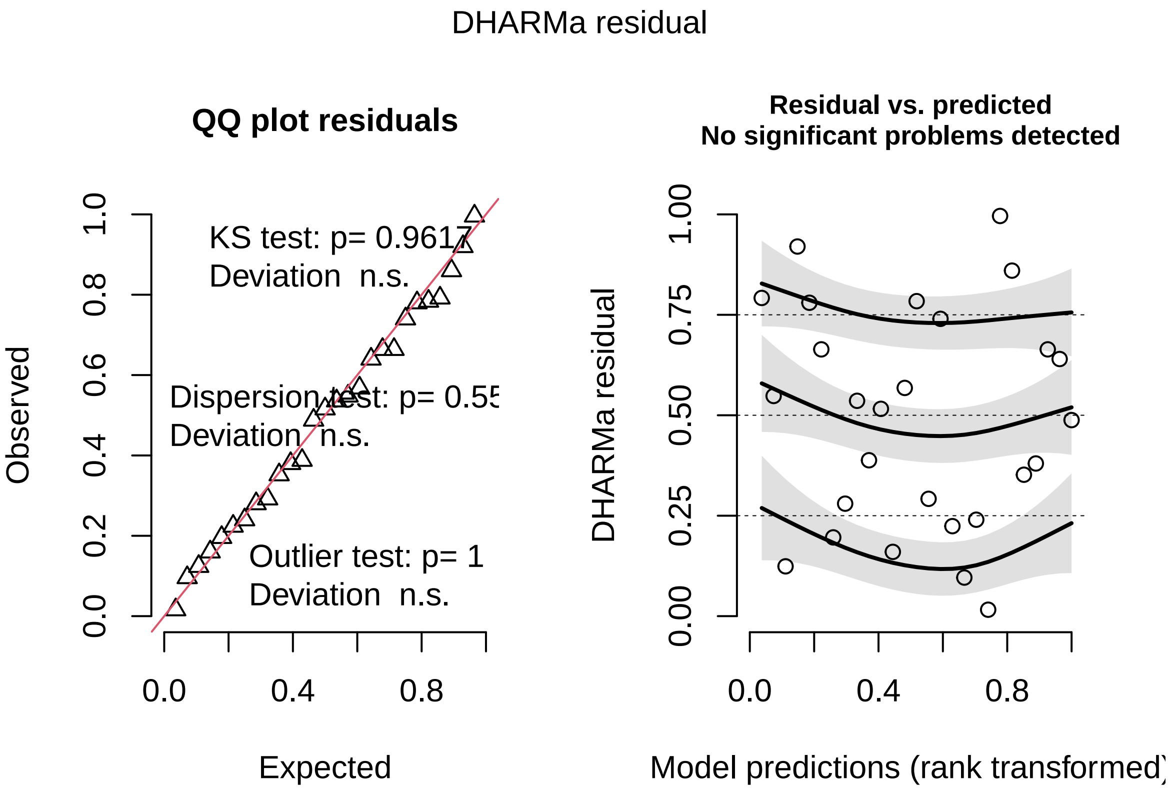

2.1.2 Model 2: Does recipient orange predict contact initiated by donors?

YES. Donors initiated fewer contacts with more orange recipients.

```
#main effects model to check variance inflation factors.
m2m <- lm(DONINITRATE ~
   +LENGTHDON
   +LENGTHREC
    +ISOREAREDREC
   +TRIALDATEJULIAN
```

```r
    +ORANGER
   +SEXDON
   +DONORWORMS,
  data = dfr2
)
car::vif(m2m)
```

```
##       LENGTHDON       LENGTHREC    ISOREAREDREC TRIALDATEJULIAN         ORANGER
##        1.080424        1.169119        1.625011        1.164438        1.737257
##          SEXDON      DONORWORMS
##        1.457674        1.159668
```

```r
m2 <- lm(DONINITRATE ~
   #+LENGTHDON
   #+LENGTHREC
    #+ISOREAREDREC
   #+TRIALDATEJULIAN
    +ORANGER,
   #+SEXDON
   #+DONORWORMS
   #+ORANGER:SEXDON
  # +ORANGER:DONORWORMS
   #+SEXDON:DONORWORMS,
  data = dfr2
)

drop1(m2)
```

```
## Single term deletions
##
## Model:
## DONINITRATE ~ +ORANGER
##         Df Sum of Sq      RSS     AIC
## <none>                0.055983 -162.82
## ORANGER  1  0.032304 0.088287 -152.52
```

```r
Anova(m2)
```

```
## Anova Table (Type II tests)
##
## Response: DONINITRATE
##             Sum Sq Df F value    Pr(>F)
## ORANGER   0.032304  1  14.426 0.0008304 ***
## Residuals 0.055983 25
## ---
## Signif. codes:  0 '***' 0.001 '**' 0.01 '*' 0.05 '.' 0.1 ' ' 1
```

```r
#visreg(m2, "ORANGER", scale="response", partial=T)
```

```r
simulateResiduals(fittedModel = m2, plot = T)
```

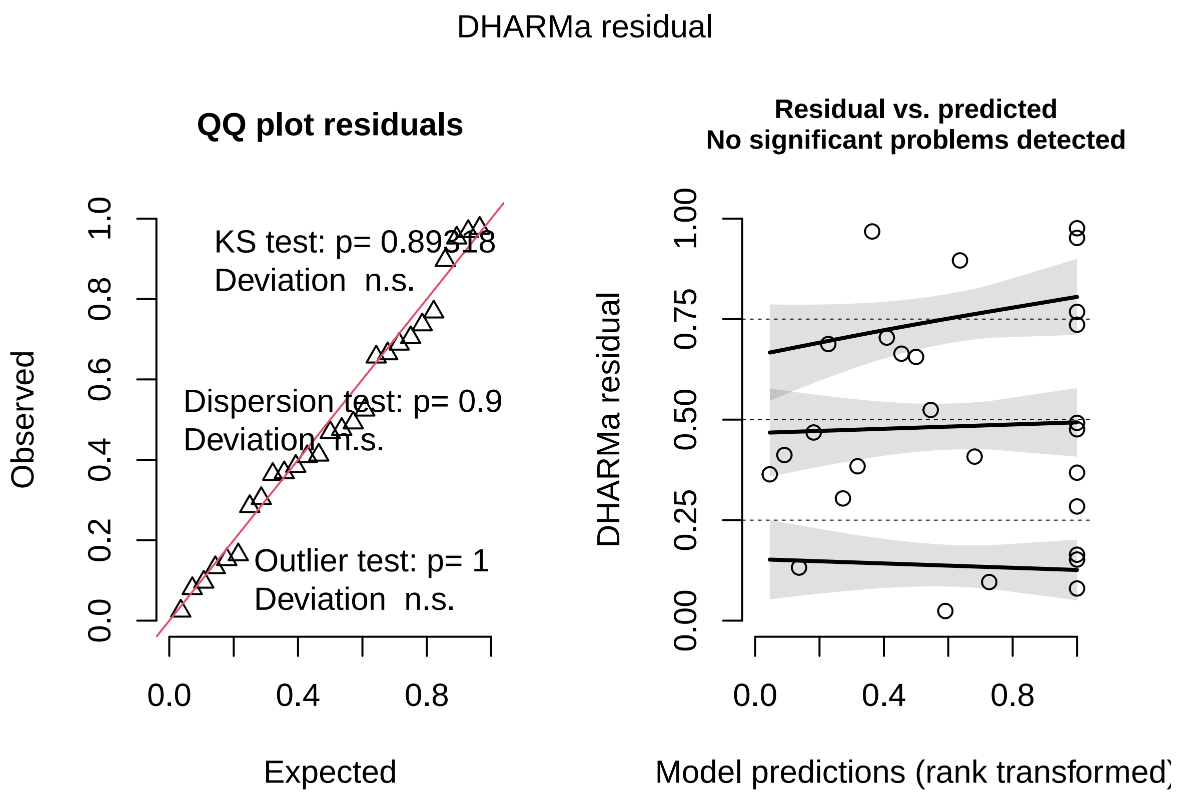

2.2 Do donor ornaments structure contact?

2.2.1 Model 3 - Does the orange on the donor structure contact initiated by the recipients?

NO. We can't explain any of the variation in recipient contact initiation rate among pairs with male donors.

```
#main effects model to check variance inflation factors.
m3m <- glm(RECINITRATE ~
   +LENGTHDON
   +LENGTHREC
    +ISOREAREDREC
   +TRIALDATEJULIAN
    +ORANGED
   +SEXREC
   +DONORWORMS,
  data = dfd
)
car::vif(m3m)
```

```
##       LENGTHDON       LENGTHREC    ISOREAREDREC TRIALDATEJULIAN         ORANGED
##        1.200865        1.173057        1.165814        1.233459        1.312686
##         SEXREC      DONORWORMS
##        1.207726        1.185227
```

```
m3 <- glm(RECINITRATE ~ 1,
   #+LENGTHDON
   #+LENGTHREC
    #+ISOREAREDREC
   #+TRIALDATEJULIAN
   # +ORANGED,
```

```
    #+SEXREC,
    #+DONORWORMS,
    #+ORANGED:SEXREC
    #+ORANGED:DONORWORMS
    #+SEXREC:DONORWORMS,
  data = dfd
)

drop1(m3)
```

```
## Single term deletions
##
## Model:
## RECINITRATE ~ 1
##        Df Deviance    AIC
## <none>      0.07234 -83.38
```

```
simulateResiduals(fittedModel = m3, plot = T)
```

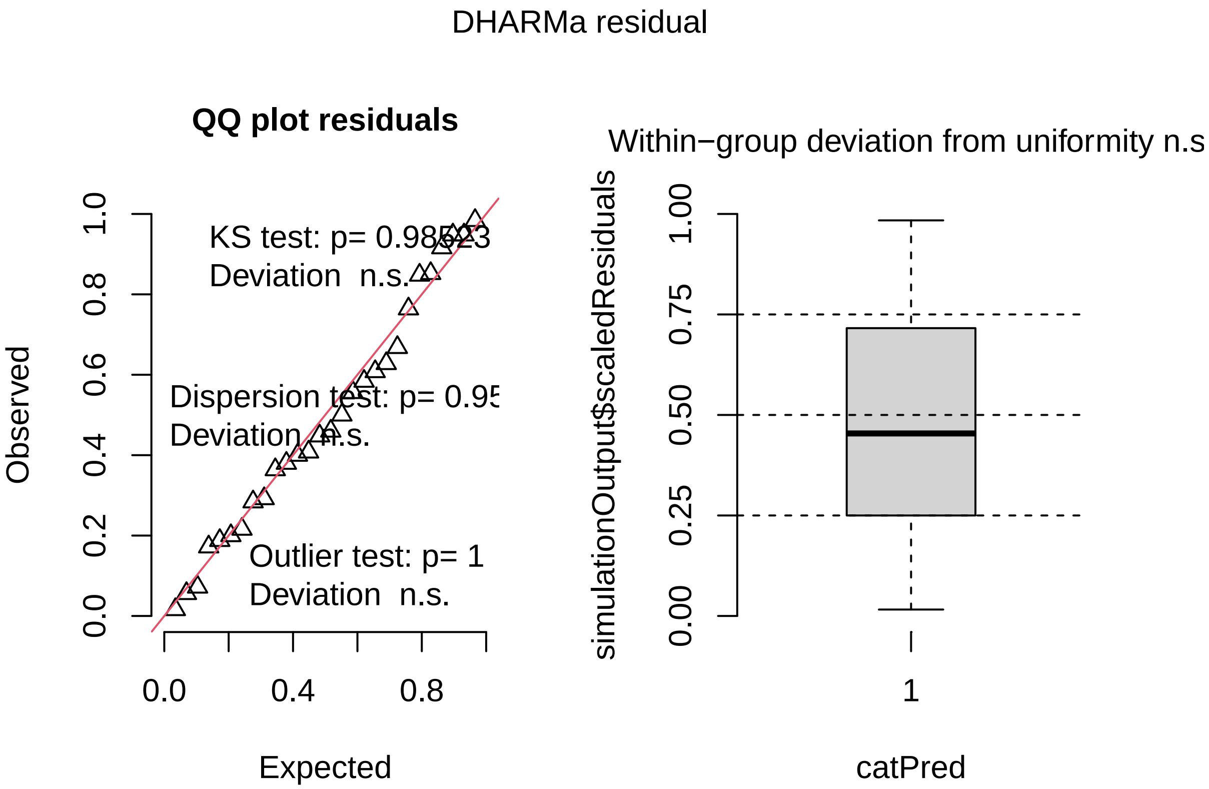

2.2.2 Model 4 - Does donor orange structure contact initiated by the donors?

When their worm loads are low, more orange donors initiate fewer contacts than less orange donors. When worm loads are high, they initiate more contacts than less orange donors.

```
#main effects model to check variance inflation factors.
m4m <- lm(DONINITRATE ~
   +LENGTHDON
   +LENGTHREC
  +ISOREAREDREC
   +TRIALDATEJULIAN
```

```
    +ORANGED
   +SEXREC
   +DONORWORMS,
  data = dfd
)
car::vif(m4m)
```

```
##       LENGTHDON       LENGTHREC    ISOREAREDREC TRIALDATEJULIAN         ORANGED
##        1.200865        1.173057        1.165814        1.233459        1.312686
##          SEXREC      DONORWORMS
##        1.207726        1.185227
```

```
m4 <- lm(DONINITRATE ~
   #+LENGTHDON
   #+LENGTHREC
 # +ISOREAREDREC
  # +TRIALDATEJULIAN
    +ORANGED
  # +SEXREC
   +DONORWORMS
  # +ORANGED:SEXREC
   +ORANGED:DONORWORMS,
  # +SEXREC:DONORWORMS,
  data = dfd
)

drop1(m4)
```

```
## Single term deletions
##
## Model:
## DONINITRATE ~ +ORANGED + DONORWORMS + ORANGED:DONORWORMS
##                    Df Sum of Sq      RSS     AIC
## <none>                          0.061058 -163.59
## ORANGED:DONORWORMS  1  0.011351 0.072409 -160.81
```

```
Anova(m4)
```

```
## Anova Table (Type II tests)
##
## Response: DONINITRATE
##                      Sum Sq Df F value  Pr(>F)
## ORANGED            0.005191  1  2.0404 0.16606
## DONORWORMS         0.001415  1  0.5560 0.46312
## ORANGED:DONORWORMS 0.011351  1  4.4618 0.04526 *
## Residuals          0.061058 24
## ---
## Signif. codes:  0 '***' 0.001 '**' 0.01 '*' 0.05 '.' 0.1 ' ' 1
```

```
#visreg(m4, "ORANGED", by="DONORWORMS")
```

```
simulateResiduals(fittedModel = m4, plot = T)
```

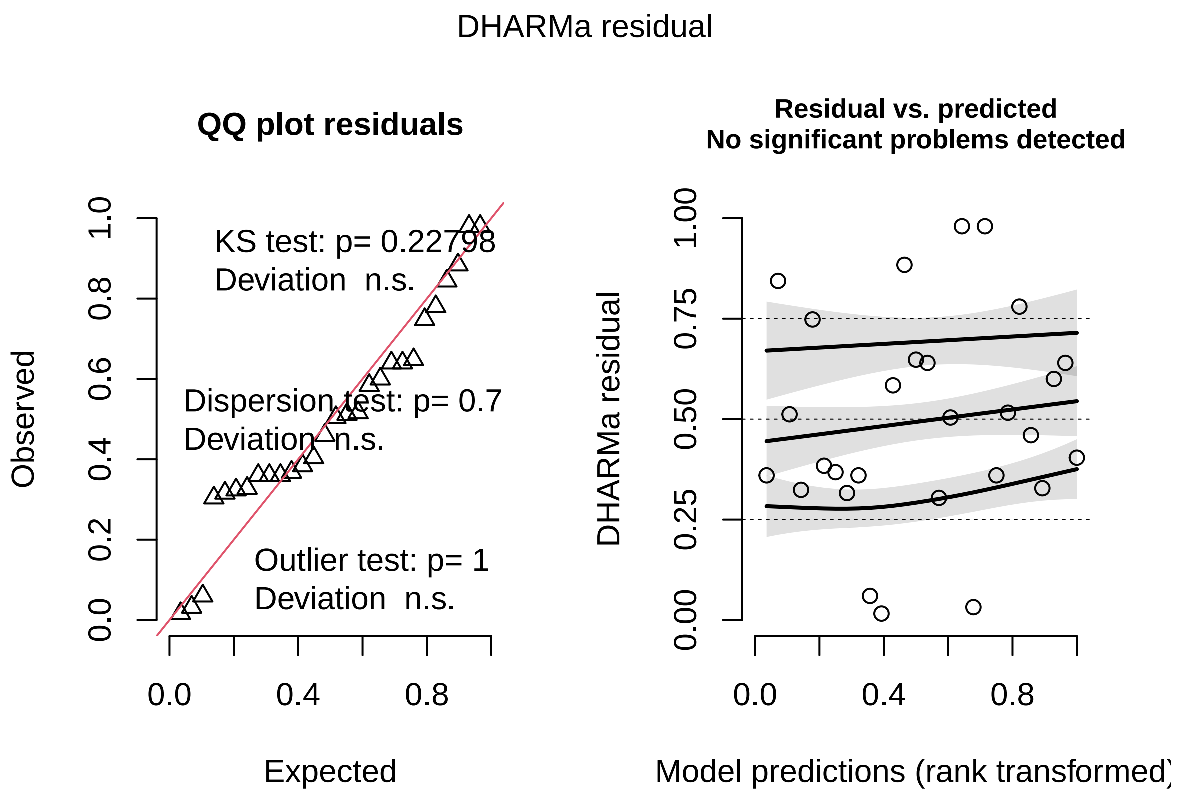

3 How does orange structure transmission?

3.1 Model 5: Do recipient ornaments structure transmission?

```
#main effects model to check variance inflation factors.
m5m <- glm.nb(TRANSWORMS1 ~
   LENGTHDON+
   LENGTHREC +
    ISOREAREDREC+
    TRIALDATEJULIAN+
    SEXDON +
    ORANGER +
    DONORWORMS +
    RECINITRATE ,
  data = dfr2,
)
car::vif(m5m)
```

```
##        LENGTHDON        LENGTHREC     ISOREAREDREC TRIALDATEJULIAN           SEXDON
##         1.164479         1.225319         1.564886        1.207268         1.326409
##          ORANGER       DONORWORMS      RECINITRATE
##         2.013364         1.156345         1.347120
```

```
m5 <- glm.nb(TRANSWORMS1 ~
    #TRIALDATEJULIAN+
    #ISOREAREDREC+
    SEXDON +
    #ORANGER +
```

```r
    DONORWORMS +
   LENGTHDON +
   #LENGTHREC +
    RECINITRATE +
    #ORANGER:RECINITRATE +
   #ORANGER:SEXDON+
    #ORANGER:DONORWORMS+
    SEXDON:RECINITRATE+
    SEXDON:DONORWORMS+
   RECINITRATE:DONORWORMS,
  data = dfr2
)

summary(m5)
```

```
##
## Call:
## glm.nb(formula = TRANSWORMS1 ~ SEXDON + DONORWORMS + LENGTHDON +
##     RECINITRATE + SEXDON:RECINITRATE + SEXDON:DONORWORMS + RECINITRATE:DONORWORMS,
##     data = dfr2, init.theta = 32272.75686, link = log)
##
## Coefficients:
##                         Estimate Std. Error z value Pr(>|z|)
## (Intercept)             -7.49050    2.10523  -3.558 0.000374 ***
## SEXDONM                  0.64524    0.81663   0.790 0.429453
## DONORWORMS               0.11285    0.02921   3.864 0.000112 ***
## LENGTHDON                0.42943    0.11897   3.610 0.000307 ***
## RECINITRATE              3.49229    6.09977   0.573 0.566965
## SEXDONM:RECINITRATE     10.86212    4.93397   2.201 0.027701 *
## SEXDONM:DONORWORMS      -0.06304    0.01420  -4.439 9.03e-06 ***
## DONORWORMS:RECINITRATE -0.36973    0.20898  -1.769 0.076853 .
## ---
## Signif. codes:  0 '***' 0.001 '**' 0.01 '*' 0.05 '.' 0.1 ' ' 1
##
## (Dispersion parameter for Negative Binomial(32272.76) family taken to be 1)
##
##     Null deviance: 102.606  on 25  degrees of freedom
## Residual deviance:  29.413  on 18  degrees of freedom
##   (1 observation deleted due to missingness)
## AIC: 121.04
##
## Number of Fisher Scoring iterations: 1
##
##
##               Theta:  32273
##           Std. Err.:  593446
## Warning while fitting theta: iteration limit reached
##
##  2 x log-likelihood:  -103.038
```

```r
Anova(m5)
```

```
## Analysis of Deviance Table (Type II tests)
##
## Response: TRANSWORMS1
```

```
##                         LR Chisq Df Pr(>Chisq)
## SEXDON                    0.0526  1   0.818671
## DONORWORMS               28.9597  1  7.390e-08 ***
## LENGTHDON                12.7239  1   0.000361 ***
## RECINITRATE               0.0453  1   0.831543
## SEXDON:RECINITRATE        4.7050  1   0.030075 *
## SEXDON:DONORWORMS        20.1170  1  7.285e-06 ***
## DONORWORMS:RECINITRATE    3.2537  1   0.071264 .
## ---
## Signif. codes:  0 '***' 0.001 '**' 0.01 '*' 0.05 '.' 0.1 ' ' 1
```

```
drop1(m5)
```

```
## Single term deletions
##
## Model:
## TRANSWORMS1 ~ SEXDON + DONORWORMS + LENGTHDON + RECINITRATE +
##     SEXDON:RECINITRATE + SEXDON:DONORWORMS + RECINITRATE:DONORWORMS
##                        Df Deviance    AIC
## <none>                      29.413 119.04
## LENGTHDON               1   42.137 129.76
## SEXDON:RECINITRATE      1   34.118 121.74
## SEXDON:DONORWORMS       1   49.530 137.16
## DONORWORMS:RECINITRATE  1   32.666 120.29
```

```
emtrends(m5, pairwise ~ SEXDON, var = "DONORWORMS")
```

```
## $emtrends
##  SEXDON DONORWORMS.trend     SE  df asymp.LCL asymp.UCL
##  F               0.06081 0.0110 Inf    0.0392    0.0824
##  M              -0.00223 0.0099 Inf   -0.0216    0.0172
##
## Confidence level used: 0.95
##
## $contrasts
##  contrast estimate     SE  df z.ratio p.value
##  F - M       0.063 0.0142 Inf   4.439  <.0001
```

```
emtrends(m5, pairwise ~ SEXDON, var = "RECINITRATE")
```

```
## $emtrends
##  SEXDON RECINITRATE.trend   SE  df asymp.LCL asymp.UCL
##  F                  -5.57 3.70 Inf   -12.810      1.68
##  M                   5.30 3.09 Inf    -0.767     11.36
##
## Confidence level used: 0.95
##
## $contrasts
##  contrast estimate   SE  df z.ratio p.value
##  F - M       -10.9 4.93 Inf  -2.201  0.0277
```

```
#visreg(m5, "ORANGER", partial=T)
#visreg(m5,"RECINITRATE",by="SEXDON", partial=T)
#visreg(m5,"DONORWORMS",by="SEXDON", partial=T, scale="response")
visreg(m5,"DONORWORMS",by="RECINITRATE", partial=T, scale="response")
```

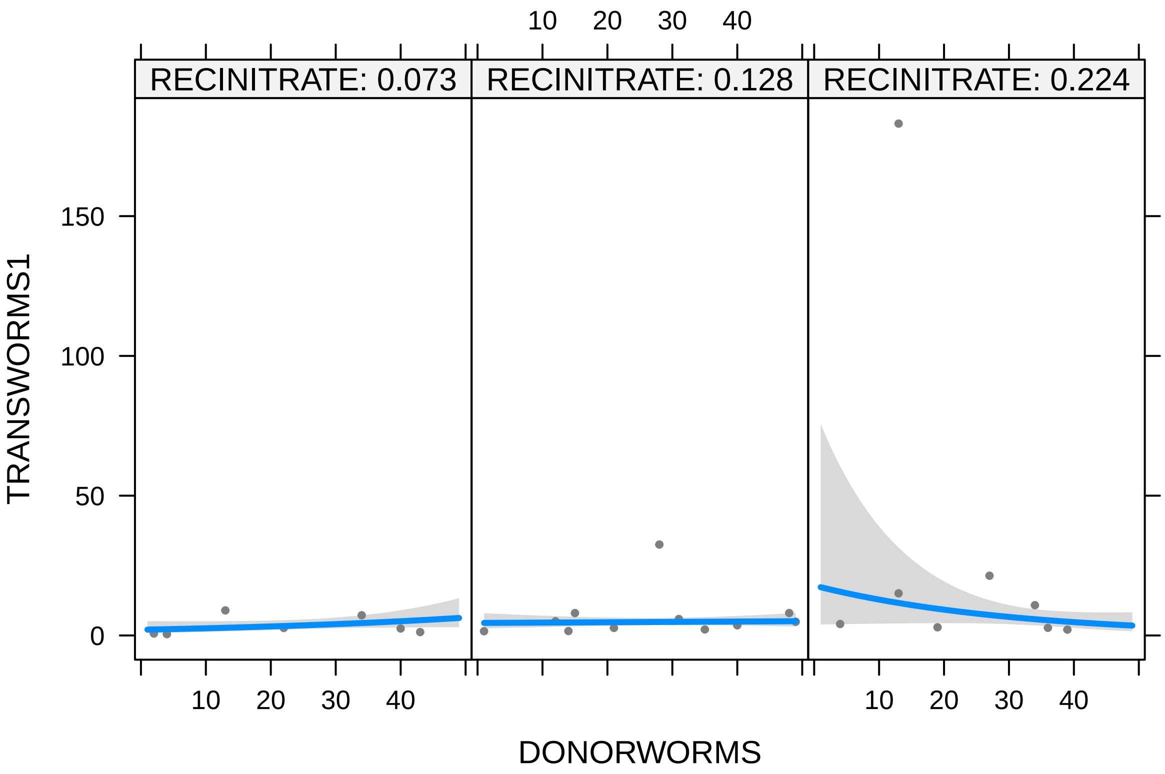


```r
visreg(m5,"LENGTHDON", partial=T, scale="linear")
```

```
## Conditions used in construction of plot
## SEXDON: M
## DONORWORMS: 24.5
## RECINITRATE: 0.1299559
```

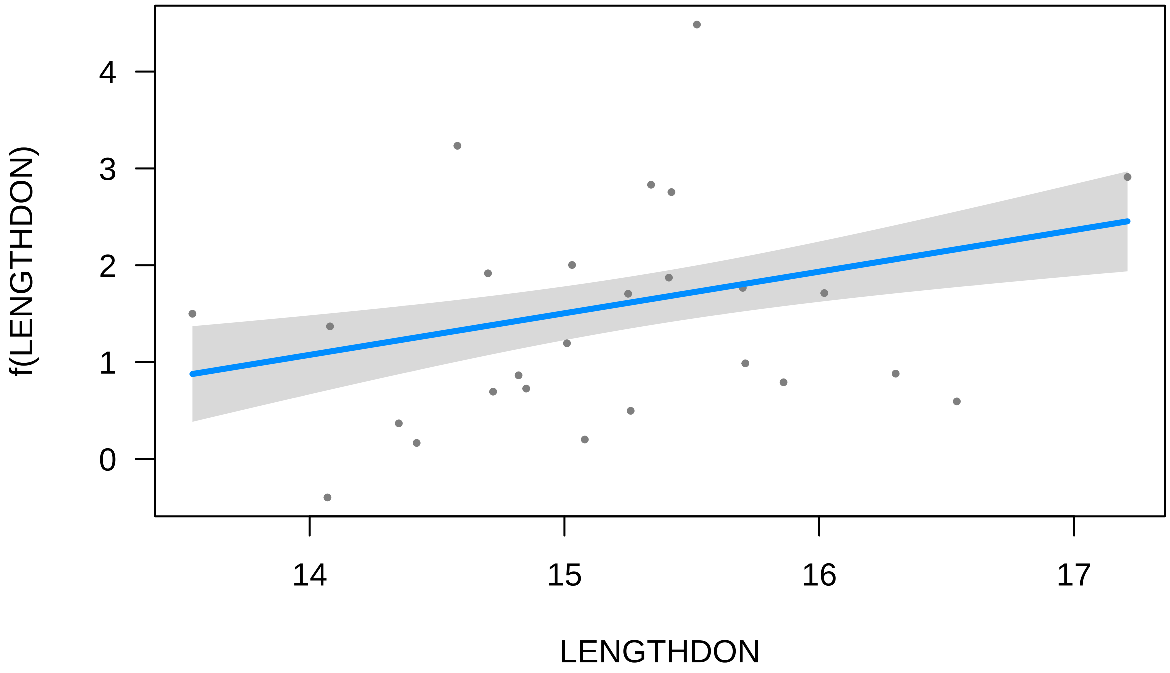


```r
simulateResiduals(fittedModel = m5, plot = T)
```

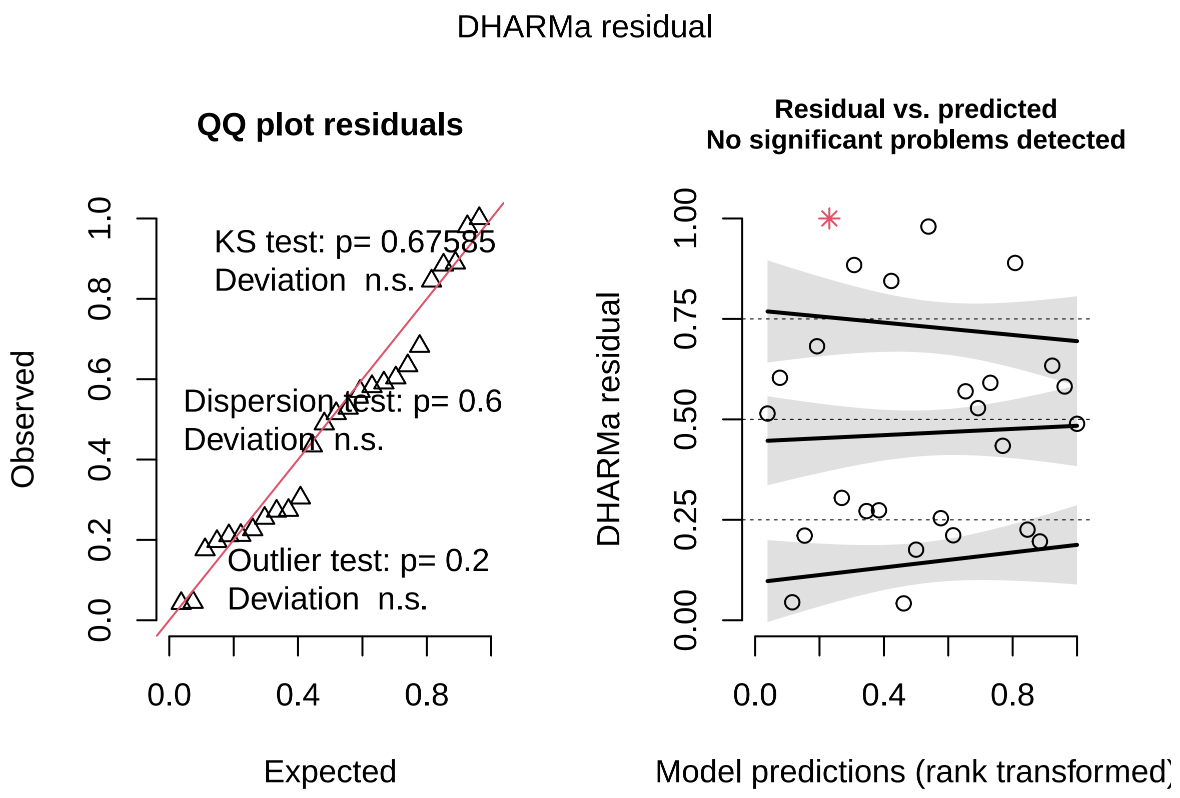

3.2 Model 6: Do donor ornaments structure transmission?

```
#main effects model to check variance inflation factors.
m6m <- glm.nb(
  TRANSWORMS1 ~
    TRIALDATEJULIAN+
    ISOREAREDREC+
    SEXREC +
    ORANGED +
    DONORWORMS +
   LENGTHDON+
   LENGTHREC +
    RECINITRATE,
  data = dfd
)
car::vif(m6m)
```

```
## TRIALDATEJULIAN    ISOREAREDREC          SEXREC         ORANGED      DONORWORMS
##        1.193205        1.285388        1.219266        1.349759        1.233994
##       LENGTHDON       LENGTHREC     RECINITRATE
##        1.401562        1.265737        1.152531
```

```
m6 <- glm.nb(
  TRANSWORMS1 ~
    #ISOREAREDREC+
    #TRIALDATEJULIAN+
    #SEXREC +
    ORANGED +
    DONORWORMS +
```

```r
   LENGTHDON+
   #LENGTHREC +
    #RECINITRATE +
    #ORANGED:SEXREC+
    ORANGED:DONORWORMS,
    #ORANGED:RECINITRATE+
    #SEXREC:RECINITRATE,
    #SEXREC:DONORWORMS+
   #RECINITRATE:DONORWORMS,
  data = dfd
)

summary(m6)
```

```
##
## Call:
## glm.nb(formula = TRANSWORMS1 ~ ORANGED + DONORWORMS + LENGTHDON +
##     ORANGED:DONORWORMS, data = dfd, init.theta = 4.447911036,
##     link = log)
##
## Coefficients:
##                     Estimate Std. Error z value Pr(>|z|)
## (Intercept)        -4.023158   2.196981  -1.831  0.06707 .
## ORANGED            -0.105130   0.073180  -1.437  0.15083
## DONORWORMS         -0.016086   0.009601  -1.675  0.09385 .
## LENGTHDON           0.378784   0.139406   2.717  0.00659 **
## ORANGED:DONORWORMS  0.007318   0.002455   2.981  0.00288 **
## ---
## Signif. codes:  0 '***' 0.001 '**' 0.01 '*' 0.05 '.' 0.1 ' ' 1
##
## (Dispersion parameter for Negative Binomial(4.4479) family taken to be 1)
##
##     Null deviance: 41.027  on 27  degrees of freedom
## Residual deviance: 28.273  on 23  degrees of freedom
## AIC: 147.86
##
## Number of Fisher Scoring iterations: 1
##
##
##               Theta:  4.45
##           Std. Err.:  2.28
##
##  2 x log-likelihood:  -135.861
```

```r
Anova(m6)
```

```
## Analysis of Deviance Table (Type II tests)
##
## Response: TRANSWORMS1
##                    LR Chisq Df Pr(>Chisq)
## ORANGED              1.6478  1   0.199258
## DONORWORMS           0.2160  1   0.642084
## LENGTHDON            7.2578  1   0.007059 **
## ORANGED:DONORWORMS   7.7436  1   0.005390 **
## ---
```

```
## Signif. codes:  0 '***' 0.001 '**' 0.01 '*' 0.05 '.' 0.1 ' ' 1
```

```r
drop1(m6)
```

```
## Single term deletions
##
## Model:
## TRANSWORMS1 ~ ORANGED + DONORWORMS + LENGTHDON + ORANGED:DONORWORMS
##                      Df Deviance    AIC
## <none>                    28.273 145.86
## LENGTHDON             1   35.531 151.12
## ORANGED:DONORWORMS    1   36.017 151.60
```

```r
#visreg(m6,"ORANGED",by="DONORWORMS", scale="response",partial=T)

visreg(m6,"LENGTHDON", scale="response",partial=T)
```

```
## Conditions used in construction of plot
## ORANGED: 2.155655
## DONORWORMS: 28.5
```

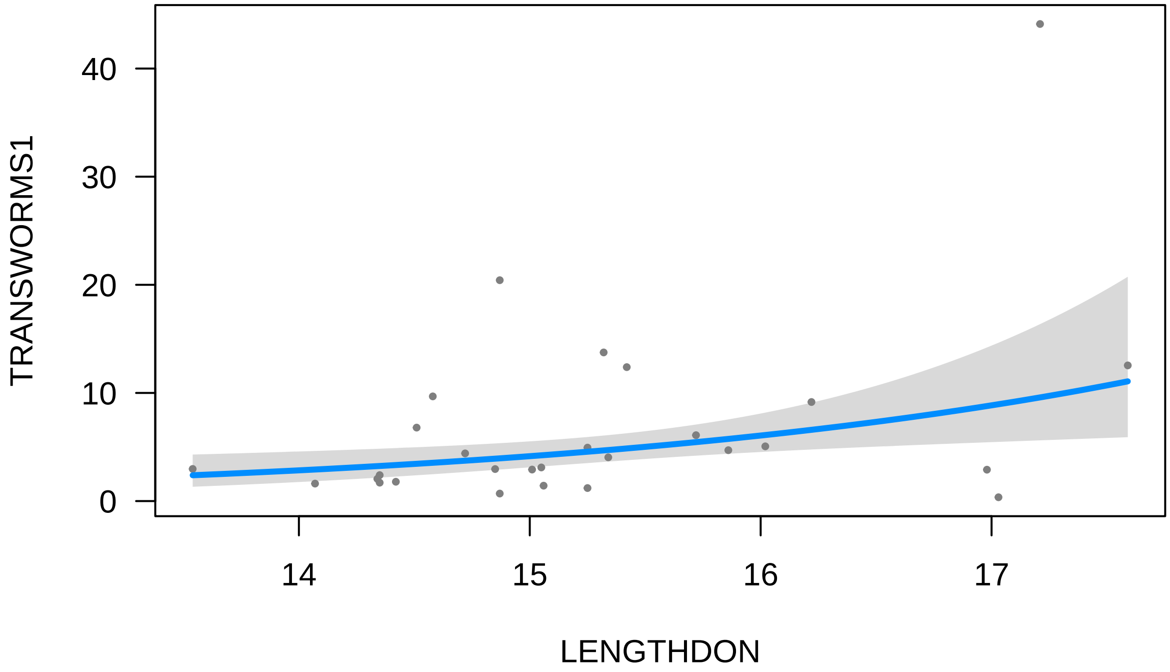


```r
simulateResiduals(fittedModel = m6, plot = T)
```

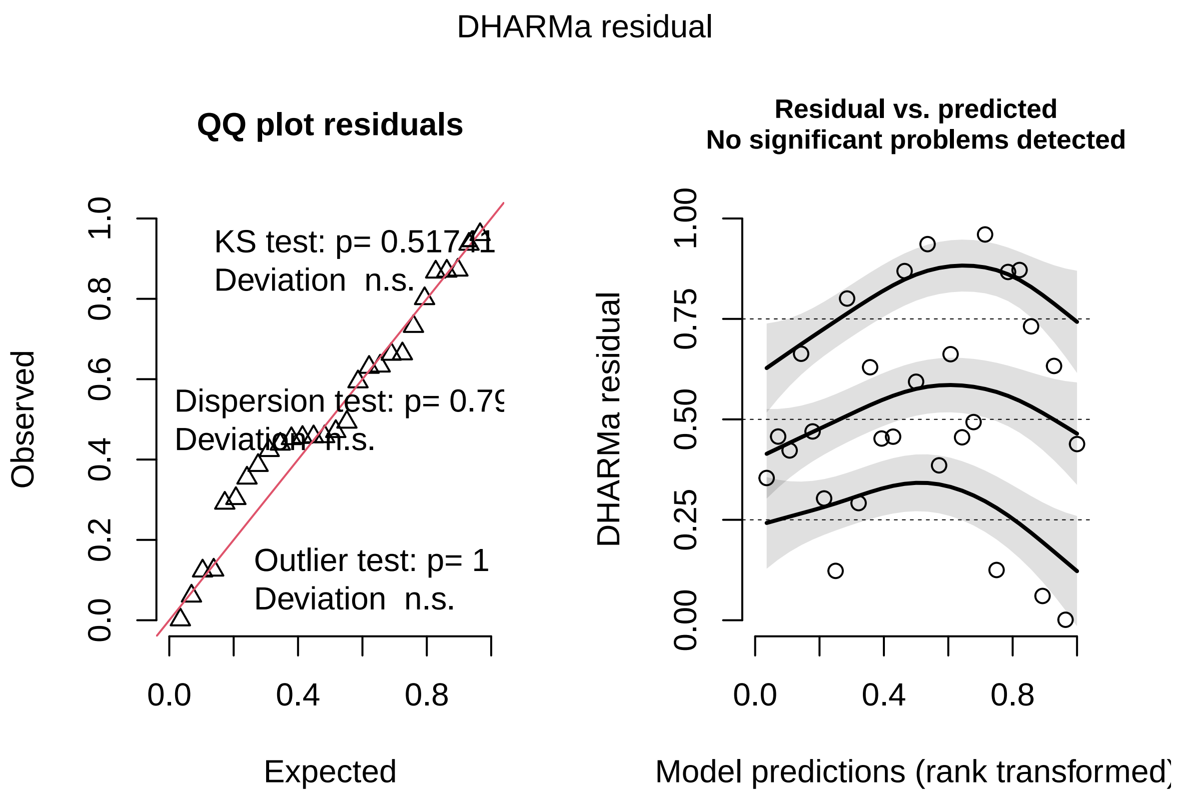

4 R and Library Versions

```r
version
```

```
##
## platform       aarch64-apple-darwin20
## arch           aarch64
## os             darwin20
## system         aarch64, darwin20
## status
## major          4
## minor          4.0
## year           2024
## month          04
## day            24
## svn rev        86474
## language       R
## version.string R version 4.4.0 (2024-04-24)
## nickname       Puppy Cup
```

```r
installed.packages()[names(sessionInfo()$otherPkgs), "Version"]
```

```
##        lme4      Matrix        MASS     emmeans   patchwork  kableExtra       knitr
## "1.1-35.3"     "1.7-0"    "7.3-64"    "1.10.1"     "1.3.2"     "1.4.0"      "1.46"
##   lubridate     forcats     stringr       dplyr       purrr       readr       tidyr
##     "1.9.4"     "1.0.0"     "1.5.1"     "1.1.4"     "1.0.2"     "2.1.5"     "1.3.1"
##      tibble   tidyverse     visreg      DHARMa         car     carData     glmmTMB
##     "3.2.1"     "2.0.0"     "2.7.0"     "0.4.6"     "3.1-2"     "3.0-5"     "1.1.9"
##      GGally     ggplot2      readxl        here
```

```
##    "2.4.0"    "4.0.0"    "1.4.3"    "1.0.1"
```

5 References

Stephenson, Jessica F, Martin Stevens, Jolyon Troscianko, and Jukka Jokela. 2020. "The Size, Symmetry, and Color Saturation of a Male Guppy's Ornaments Forecast His Resistance to Parasites." *Am. Nat.* 196 (November): 597–608.
